# Knowledge, attitudes, and practices of mothers regarding childhood malaria in southeastern Gabon

**DOI:** 10.1186/s12936-023-04584-5

**Published:** 2023-05-15

**Authors:** Judicaël Boris Lendongo Wombo, Chérone Nancy Mbani Mpega Ntigui, Lydie Sandrine Oyegue-Liabagui, Euloge Ibinga, Sydney Maghendji-Nzondo, Franck Mounioko, Steede Seinnat Ontoua, Alain Prince Okouga, Jean Bernard Lekana-Douki, Edgard Brice Ngoungou

**Affiliations:** 1 Unit of Research in Health Ecology, Interdisciplinary Centre for Medical Research of Franceville (CIRMF), Franceville, Gabon; 2 Département d’Epidémiologie, Biostatistiques et Informatique Médicale (DEBIM), Unité de Recherche en Epidémiologie des Maladies Transmissibles Santé Environnement (UREMSCE), Université des Sciences de la Santé (USS), Owendo, 4009 BP Gabon; 3 Ecole Doctorale Régionale d’Afrique Centrale en Infectiologie Tropicale (ECODRAC), Franceville, Gabon; 4 Department of Biology, Faculty of Science, Masuku University of Science and Technology (USTM), Franceville, Gabon; 5 Department of Parasitology, Health Science University (USS), Owendo, Libreville, 4009 BP Gabon

**Keywords:** Malaria knowledge, Malaria perception, Malaria practices, Maternal perception, Community-based management

## Abstract

**Background:**

In Gabon, children under 5 years of age and pregnant women are the populations who are most at risk of malaria. Despite the presence of accessible health facilities, the community-based management of childhood fever remains a very common practice in Gabon, which may have serious consequences on child health. As such, the objective of this descriptive cross-sectional survey is to assess the mothers’ perception and knowledge of malaria and its severity.

**Methods:**

Different households were selected using the simple random sampling method.

**Results:**

A total of 146 mothers from different households were interviewed in the city of Franceville, in southern Gabon. Among the households interviewed, 75.3% had a low monthly income (below the minimum monthly income of $272.73). Among the respondents, 98.6% of mothers had heard of malaria and 55.5% had heard of severe malaria. Regarding preventive measures, 83.6% of mothers used an insecticide-treated net as a means of protection. Self-medication was practiced by 68.5% of women (100/146).

**Discussion:**

The use of health facilities was motivated by better care, the decision of the head of the family, but above all by the severity of the disease. Women identified fever as the main symptom of malaria, which could be beneficial for a quicker and more efficient management of the disease in children. Malaria educational campaigns should also increase awareness of severe forms of malaria and its manifestations. This study shows that Gabonese mothers react quickly when their children have fever. However, several external factors lead them to practice self-medication as a first resort. In this survey population, the practice of self-medication did not depend on social status, marital status, level of education, on the young age or inexperience of mothers (p > 0.05).

**Conclusions:**

The data revealed that mothers may underestimate severe malaria and delay medical care by self-medicating, which can have detrimental effects for children and hinder the regression of the disease.

## Background

To date, malaria remains the most prevalent vector-borne parasitic disease in the world and a major public health problem in sub-Saharan Africa. Among the 241 million malaria cases and 627,000 deaths worldwide in 2020, sub-Saharan Africa accounted for more than 96% of cases [1]. Malaria is both a disease of poverty and a cause of poverty [2]. Delayed urbanization, poor quality of life, underfunded and poorly managed health services with long consultation times, all make malaria eradication difficult. Despite the high intensity of malaria infection in the African region, the use of control measures is still often insufficient in developing countries. Furthermore, people only consult health services when their practice of self-medication has failed despite the availability of early diagnosis and treatment for malaria in health facilities [3–5].

According to the World Health Organization (WHO), the majority of these reported cases in sub-Saharan Africa are children under 5 years of age which account for 67% of cases [1]. Moreover, in the Central African region, malaria is one of the main causes of morbidity and mortality for vulnerable groups such as pregnant women and young children [6–8]. In children, malaria can cause several organ malfunctions, which can lead to complicated forms of the disease. Severe anaemia is one of the main manifestations of severe malaria in children aged under five, followed by cerebral malaria and respiratory distress [9–11].

In Gabon, a central African country, malaria transmission is stable and intense throughout the year. According to the National Malaria Control Programme (NMCP), Gabon has recorded nearly 799,000 cases of malaria with over 500 deaths. Yet, in 2010, the country had 50% coverage of insecticide-treated nets (ITNs) and 99% coverage of artemisinin-based combination therapy (ACT). However, in recent years, the absence of the NMCP in the field and, therefore, the absence of awareness campaigns, education on malaria epidemiology and the distribution or sale of ITNs are becoming increasingly rarer each year [12].

The city of Franceville, located in southeastern Gabon, is the capital of the Haut-Ogooué province and the third largest city in the country. This city has a high population but mostly with a low economic status. Franceville is an urban area but additional urbanization efforts are needed as anarchic constructions favor the presence of mosquito breeding grounds and, therefore, their proliferation. The prevalence rate of malaria in Franceville is approximately 20% and the most frequently encountered plasmodial species is *Plasmodium falciparum*.

Despite the accessibility of health facilities, the community-based management of childhood fever remains a very common practice in Gabon and leads to a delay in proper care by health facilities. The objective of this study was to evaluate the level of knowledge of the various symptoms of childhood malaria among mothers, as well as their attitudes and care practices.

## Methods

### Type of study and location

This study is a descriptive cross-sectional survey. It was conducted in six districts (Ongouégné, Sable, Alélé, Ombelé, Ongali, Yéné) of the city of Franceville, in southeastern Gabon. The study was conducted during the month of June 2021. The different households were selected by the simple random sampling method. The city of Franceville is an administrative city whose neighborhoods are mostly under-integrated. According to the latest population census, dating from 2013, the city of Franceville had 129,694 inhabitants [13].

### Data collection

Mothers from each selected household were included in the study after obtaining their informed consent. A survey with closed-ended questions was administered through an interview. The structured interviews were conducted by a team of two examiners. The information collected from the mothers in each household included socio-demographic data; knowledge, attitudes, and practices about malaria and its different forms; use of preventive or control means for malaria; possession of ITNs; practice of self-medication and type of medication used. The survey of mothers was conducted in French for those who spoke French and in the vernacular language of the region (Teke-Obamba) for those who did not speak French well.

### Data analysis

The different answers of all interviewed mothers were recorded in Excel 2013 spreadsheets. Statistical analyses were performed with Epi-info version 3.3.2 (2005, CDC, Atlanta, USA) and RStudio version software (version 1.1.419). Categorical variables were described by proportions. These proportions were compared using the Chi2 test. For all analyses, the significance level was set at α = 5%.

Ethical clearance and approval was obtained from the National Research Ethics Committee in Gabon (No. 001/PR/SG/CNER/2018). Informed written consents were obtained from all levels of local government prior to data collection, and verbal consents were obtained from participants during data collection. Respondents were given the right to refuse or participate in the study and to withdraw from the study at any time. Privacy and confidentiality were maintained throughout the study.

## Results

### Socio-demographic characteristics of mothers

A total of 146 mothers from different households were interviewed in the city of Franceville. All of the households visited were from under-integrated neighbourhoods (100%). The Ongouégné neighbourhood had the highest proportion of mothers interviewed (30.3%), followed by the Alélé neighbourhood (29.4%). The majority of the study population had a middle school education (55.5%), followed by primary (Table 1) education (34.2%). Approximately 75.3% of the households surveyed had a low monthly income (below the minimum monthly income of about 272 dollars). The majority of mothers were unemployed (54.8%), and 15.7% were students. The study population was predominantly composed of young mothers, as 54.8% of women were aged 18 to 30 years.

### Description of the mothers’ knowledge of malaria

The analysis of the mothers’ knowledge showed that 98.6% had at least heard of malaria and 55.5% of them knew about severe malaria. Among the 146 mothers, 93.2% cited mosquito as the vector of malaria and 83.6% of them used ITNs as a means of protection. Other information is shown in Table 2.

**Table 1:** Socio-demographic characteristics of the mothers in the study

characteristics	Modality	Numbers	%
Age group (in years) of mothers	[18–30]	80	54.8
	[31–40]	47	32.2
	[41–50]	13	8.9
	> 50	6	4.1
Marital status	Married	19	13
	Cohabitation	59	40.4
	Single	67	45.9
	Divorced	1	0.7
Level of education	Primary school	50	34.2
	Middle school	81	55.5
	High school	15	10.3
Mother’s occupation	Health Officer	7	4.8
	Security officer	1	0.7
	Merchant	16	10,9
	Student	23	15.7
	University student	6	4.1
	Surface technician	3	2.1
	Teacher	6	4.1
	Mecanist	1	0.7
	Secretary	1	0.7
	Drugstore saleswoman	2	1.4
	Unemployed	80	54.8
Economic/household level	Low	110	75.3
	Mean	11	7.5
	High	25	17.2
Number of people per household	[1–3]	33	22.6
	[4–6]	66	45.2
	[7–10]	36	24.7
	> 10	11	7.5
Place of residence	Ongouégné	44	30.3
	Sable	17	11.6
	Alélé	43	29.4
	Ombelé	13	8.9
	Ongali	15	10.3
	Yéné	14	9.5

**Table 2:** Mothers’ knowledge of malaria in Franceville, Gabon

Questions	Participant Responses	Number	%
Knowledge of malaria	Yes	144	98.6
	No	2	1.4
Symptoms in children	Fever	125	85.6
	Vomiting	71	48.6
	Chills	44	30.1
	Diarrhea	22	15.1
	Température > 37.5 °C	81	55.5
	Other^*^	106	72.6
Knowledge of severe malaria	Yes	81	55.5
	No	65	44.5
Signs of severe malaria	Neuromalaria	34	23.3
	Convulsions	67	45.9
	Severe anemia	50	34.2
	Respiratory distress	24	16.4
	Prostration	27	18.5
	Other**	20	13.7
Sources of information	Television	11	7.5
	Radio	4	2.8
	Hospital	45	30.8
	Awareness campaign	13	8.9
	Community relay	73	50
Vectors	Mosquitoes	136	93.2
	Does not know	9	6.2
	Other	1	0.7
Preventives measures	ITNs	122	83.6
	IDP	41	28.1
	Other^***^	94	64.4
Factors favoring the development of mosquitoes	Unsanitary conditions	100	68.5
	Poverty	7	4.8
	Stagnant water	94	64.4

*(e.g., headache, stomach ache, fatigue, cough); **(altered consciousness, hypoglycemia, fatigue, diarrhea); ***(fan, hard house, drinking alcohol). ITNs: insecticide-treated bed nets, IDP: intra-domiciliary pulverization

**Table 3:** Determinants of the mothers’ self-medication practices

Determinants	Modality	Number	%	P
Age group of mothers (in years)	[18–30]	52	52	0.21
	[31–40]	34	34	
	[41–50]	8	8	
	> 50	4	4	
Level of education	Primary school	37	37	0.2
	Middle school	56	56	
	Higher school	7	7	
Marital status	Maried	14	14	0.19
	Cohabiting	41	41	
	Single	45	45	
Household standard of living	Low	78	78	0.19
	Mean	7	7	
	High	15	15	

### Assessment of the mothers’ attitudes and practices

**Fig. 1:**
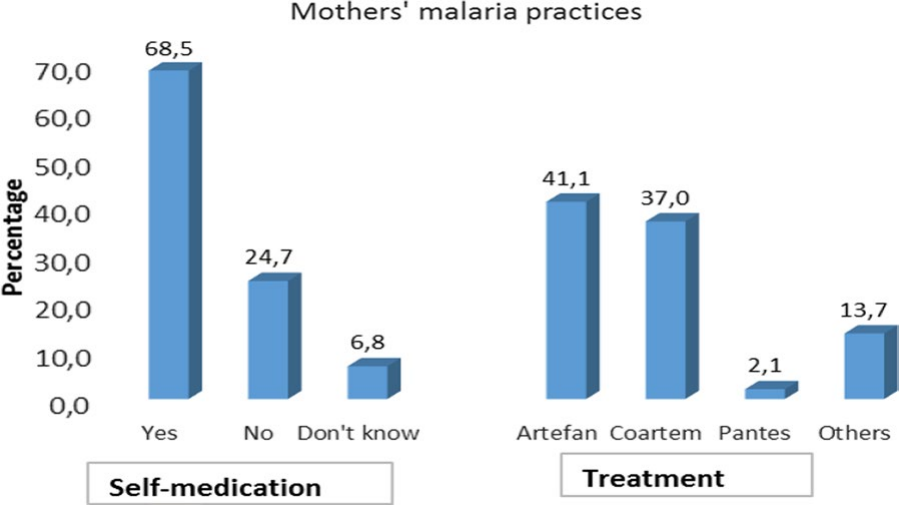
Malaria pratices of mothers in Franceville, Gabon

The results show that 68.5% (100/146) of mothers chose to self-medicate when their child had malaria and only 24.7% (36/146) of women went to a health centre as is proper. The use of health facilities was motivated by better (Table 3) care, the decision of the head of the family and, to a lesser extent, the severity of the disease. During the onset of fever in children, the practice of self-medication consisted of buying medicines in pharmacies and administering mainly Artefan (41.1%, 60/137), followed by Coartem (37.0%, 54/137). All the information is recorded in Fig. 1.

### Comparison of self-medication by socio-demographic data

The analysis of these data showed that the practice of self-medication was not related to age, level of education, marital status and standard of living of the mother’s household in relation to the occurrence of malaria in children (p > 0.05).

## Discussion

This study highlights the unfavorable socio-economic conditions to which the majority of mothers (75.3%) are subjected in their households in Franceville. Given that malaria is a disease which affects impoverished areas [14], the study was conducted to focus on sub-integrated neighbourhoods of Franceville to conduct this study.

All of the mothers who participated in the study had attended school (100%) and 98.6% of them had knowledge of the epidemiology of malaria and its mode of transmission (93.2%). The proportion of women with a knowledge of the mode of transmission was higher than that in other studies, 17.3% and 63% in Ethiopia [15, 16]. In this study population, 93.2% of women had good knowledge of the malaria vector, and 85.6% named fever as the main symptom of malaria. Other studies showed that mothers in Togo and Senegal also identify fever as the main symptom of malaria, with proportions of 72% and 81%, respectively [17, 18]. Knowledge of fever as the main symptom of malaria among mothers is beneficial for a rapid and effective management of the disease in children. However, the findings suggest that educational campaigns should also insist on the manifestations of severe forms of malaria. In fact, only 55.5% of the population surveyed in our study had knowledge of severe malaria and its different clinical signs.

The main sources of information for the surveyed population were community relays (50.0%), followed by health agents (30.8%) and malaria awareness campaigns (8.9%). These results are partially similar to those of Seck et al. in Senegal [18], where the dominant source of information were community health workers (62.9%).

These findings show that mothers react quickly to fever in their children. However, several external factors lead them to self-medicate as a first resort. This practice has also been widely reported in other studies focused on mothers with febrile children, such as in Tunisia with a frequency of 88.2%. In a study conducted in Gabon, 24.1% of mothers practiced self-medication [19–21]. This practice of self-medication could be a major problem by leading to the emergence of strains resistant to the different anti-malarial drugs. In this study population, the practice of self-medication did not depend on social status, marital status, level of education, on the young age or inexperience of mothers. This result could be explained by the fact that mothers are very responsive when their child has a fever, and even better, they always have antipyretics in reserve at home to bring down the child’s temperature in case of fever.

## Conclusion

This descriptive cross-sectional study was conducted to support education campaigns and health measures to control malaria in Franceville, Gabon. The data revealed that mothers may underestimate severe malaria and delay medical care by self-medicating, which can have detrimental effects for children and hinder the regression of the disease. These results show that there is a need for the Ministry of Health through the NMCP to increase awareness campaigns on severe malaria in Gabon so that the population can improve their knowledge of the disease and seek medical care.

## Data Availability

The datasets used and/or analysed during the current study available from the corresponding author on reasonable request.

## Abbreviations

WHO: World Health Organization
ITNs: Insecticide-Treated bed Net
ACT: Artemisinin-based Combination Therapy
